# A novel fluorescence immunochromatographic assay strip for the diagnosis of schistosomiasis japonica

**DOI:** 10.1186/s13071-020-04511-6

**Published:** 2021-01-06

**Authors:** Yuanxi Shen, Rongyi Ji, Rui Chai, Nana Yuan, Jiyue Zhang, Yi Jing, Man Yang, Lanqi Zhang, Yang Hong, Jiaojiao Lin, Chuangang Zhu

**Affiliations:** 1 Key Laboratory of Animal Parasitology, Ministry of Agriculture of China, Shanghai Veterinary Research Institute, Chinese Academy of Agricultural Sciences, Shanghai, China; 2 Jiangsu Co-innovation Center for Prevention and Control of Important Animal Infectious Diseases and Zoonoses, Yangzhou, China; 3 University of Reading, Whiteknights, Reading, Berkshire RG26UA England

**Keywords:** Fluorescence immunochromatographic assay, ELISA, *Schistosoma japonicum*

## Abstract

**Background:**

Schistosomiasis japonica is a severe zoonosis. Domestic animals are the primary source of infection and play an important role in disease transmission. Surveillance and diagnosis play key roles in schistosomiasis control; however, current techniques for the surveillance and diagnosis of the disease have limitations. In this study, we developed a novel fluorescence immunochromatographic assay (FICA) strip to detect anti-*Schistosoma japonicum* antibodies in host serum.

**Methods:**

A FICA strip was developed for the diagnosis of *Schistosoma japonicum* in domestic animals. Streptococcus protein G (SPG) and soluble egg antigen (SEA) were transferred onto a nitrocellulose (NC) membrane to form the control line (C) and the test line (T), respectively. With fluorescence activity as well as binding activity to multispecies IgG, the recombinant protein rSPG-RFP was expressed and employed as an antibody indicator in the FICA strips.

**Results:**

The dual gene fusion plasmid was verified by PCR and restriction enzyme digestion. The expressed recombinant protein was 39.72 kDa in size, which was consistent with the predicted molecular weight. The western blot results showed binding activity between rSPG-RFP and IgGs from different hosts. Fluorescence microscopy also showed the fluorescence activity of the protein present. The affinity constant (Ka) values of rSPG-RFP with rabbit, donkey, mouse and goat IgG were 1.9 × 10^5^, 4.1 × 10^5^, 1.7 × 10^5^ and 5.4 × 10^5^, respectively. Moreover, based on the recombinant protein, the test strip for detecting *S. japonicum* in buffaloes could distinguish positive from negative serum. The lower limit of detection of the FICA strip was 1:10,000. Compared with ELISA, the FICA strips exhibited similar results in the diagnosis of infection in clinical bovine serum samples, with a kappa value of 0.9660 and *P* < 0.01. The cross-reactivities of the FICA strips with *Haemonchus contortus* and *Schistosoma turkestanicum* (30.15% and 91.66%, respectively) were higher than those of ELISA (26.98% and 87.5%, respectively).

**Conclusions:**

Based on the rSPG-RFP protein that we developed, strip detection can be completed within 15 min. Heightened sensitivity allows the strip to accurately identify schistosome antibodies in serum. In conclusion, this method is convenient, feasible, rapid and effective for detecting *S. japonicum*.
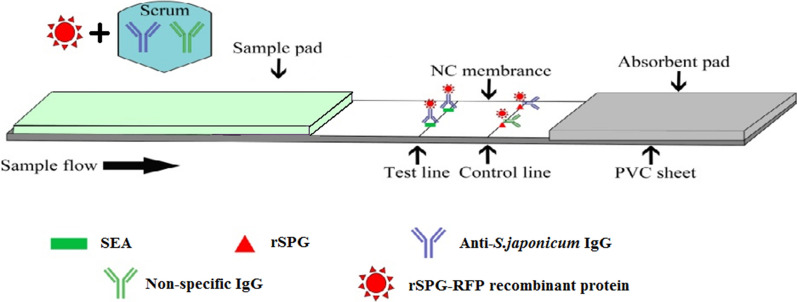

## Background

Schistosomiasis is one of the most serious zoonoses caused by schistosomes, and it remains a major public health problem worldwide. Schistosomes can infect both humans and animals, affecting the health of people and livestock and causing enormous economic losses. In addition to the chronic morbidities associated with impaired child growth and development, chronic inflammation, anemia and other nutritional deficiencies, some new disease burden assessments estimate that schistosomiasis accounts for up to 70 million disability-adjusted life years (DALYs) lost annually. This global burden estimate exceeds those for malaria and tuberculosis and is almost equivalent to the DALYs lost due to HIV/AIDS [1, 2]. Research has shown a close relationship between schistosome infection in domestic animals and humans, which indicates that when there is a significant increase in the *Schistosoma japonicum* prevalence rate in domestic animals (among which water buffalo is the key reservoir host), an increase in the human infection rate also occurs [3]. If the prevalence rate of schistosomiasis is not monitored properly, surveillance of the disease, let alone its elimination, will be difficult to achieve [4]. Therefore, the detection of *S. japonicum* in infected domestic animals is critical for the control of this disease [5].

Schistosomiasis diagnostic methods include direct parasitological observations (parasite egg detection and miracidium hatching) and indirect serological techniques (antibodies or circulating antigen detection in serum) [6]. Microscopic examination of stool (e.g., the Kato-Katz method and miracidium hatching tests) is considered the gold standard for the detection of schistosomiasis [7]. However, these procedures are time consuming and have limited sensitivity due to great day-to-day fluctuations in egg output [8–10], which is not conducive to early diagnosis.

Immunodiagnostic assays, including the test strip assay, enzyme-linked immunosorbent assay (ELISA) and indirect hemagglutination assay (IHA), have high sensitivity and specificity. Nonetheless, ELISA has some shortcomings, such as being time consuming and requiring special equipment and reagents, which make it unsuitable for field screening [11]. IHA has high sensitivity, but the interpretation of the results is subjective. The test strip assay is rapid and convenient, and the results can be read more objectively, which makes it suitable for mass field screening.

Conventional test strips for the diagnosis of schistosomiasis include the colloidal gold immunochromatographic assay (GICA) and the dot-immunogold filtration assay (DIGFA). Since the development of the rapid strip assay, it has become the first choice for the rapid diagnosis of schistosomiasis because of its sensitivity, specificity and convenience. However, the immunochromatographic test strip can be used only for qualitative detection. Additionally, the interpretation of the results is subjective. In contrast, the fluorescence immunochromatographic assay (FICA) test strip can be used for qualitative or semiquantitative detection with high sensitivity.

In this article, a recombinant protein with both IgG binding activity and fluorescence activity was developed. The soluble egg antigen (SEA) of *S. japonicum* was employed as the diagnostic antigen to establish a novel FICA.

## Materials and methods

### Serum samples

All serum samples were stored in the laboratory.

A total of 127 buffaloes were artificially infected with *S. japonicum*, with positive fecal examinations (hatching method). All buffaloes were killed 6 weeks post-challenge by portal perfusion. Investigation of the infection intensity was performed in infected buffaloes under 3% sodium pentobarbital anesthesia [3.9–4.2 ml/kg body weight (BW) or 117–126 mg/kg BW]. The eyelid radiation, pupil dilatation, muscle relaxation and heart rate of the anesthetized buffaloes were strictly monitored. Surgery started only when the buffaloes were confirmed to have died. Serum samples that were negative for *S. japonicum* were collected from 82 buffaloes that had not been artificially infected, and the fecal examination results were all negative. Forty-eight goats were naturally infected with *S. turkestanicum* according to positive fecal examinations (hatching method) conducted in Nimu County, Tibet. Sixty-three serum samples were collected from *Haemonchus contortus*-positive goats in which the parasites were found in the abomasa.

### Cloning and expression of rSPG-RFP

Primer5 was employed to design primers to amplify the sequence of the C3 domain and the red fluorescent protein (RFP) sequence from plasmids. C3 and RFP fragments were amplified from plasmids containing the streptococcal protein G (SPG) or RFP gene (the primers are shown in Table 1). After validation with the theoretical fragment size, double-enzyme digestion was conducted simultaneously on the amplified C3 and RFP fragments and the blank PET-28a(+) plasmids. QuickCut BamH1 and EcoR1enzymes (Takara, Japan) were added, and the reaction was allowed to proceed for 30 min at 16 °C. The C3 and RFP fragments and plasmids recovered from the double-enzyme digestion were ligated at a ratio of 10:1 at 37 °C for 1 h. The constructed plasmids were subsequently transformed into DH5α cells to confirm the correct ligation of C3 and RFP fragments with PCR and sequencing validation. The plasmids were then transformed into *E. coli* BL21 (DE3) cells. rSPG-RFP expression was induced, and the expressed protein was purified and analyzed by SDS-PAGE.

**Table 1:** Sequences of the primers and cleavage sites

Primers (cutting sites)	Sequence
C3 forward primer (BamH1)	CGCGGATCCACCTACAAAC
C3 reverse primer (EcoR1)	CCGGAATTCGCTACCG
RFP forward primer (EcoR1)	CCGGAATTCGCCTCCTCCGAGAAC
RFP reverse primer (Xho1)	GGCCTCGAGCTACAGGAACAG

Underline text is corresponding to the cutting sites

### Analysis of rSPG-RFP

Western blotting was employed to qualitatively identify the binding activity of rSPG-RFP by using HRP-conjugated IgG from different animals (i.e., rabbit, mouse, goat and donkey). rSPG-RFP was electrophoresed and then transferred to nitrocellulose membranes using a wet western blotting system. After blocking with 5% nonfat milk in PBS containing 0.05% Tween (PBST), the membrane was incubated with HRP-conjugated multispecies IgG (i.e., rabbit, goat, donkey and mouse). Diaminobenzidine (DAB) was used for color development.

To quantitatively determine the affinity constant (Ka) of rSPG-RFP with IgG from different animals, microtiter plates (CoStar, Acton, MA, USA) coated with rSPG-RFP at different concentrations were incubated overnight at 4 °C. Subsequently, HRP-conjugated goat anti-mouse IgG, mouse anti-rabbit IgG, donkey anti-goat IgG and rabbit anti-goat IgG were serially diluted with PBST to bind with the antigens. 3,3′,5,5′-Tetramethyl benzidine dihydrochloride (TMB) was added to the plates, and the reaction was stopped after 10 min using 2 M sulfuric acid. The OD of the wells at 450 nm was measured using a microplate reader (Tecan, Mannedorf, Switzerland), using the OD measured at 450 nm as the ordinate and the logarithm of the antibody concentration as the abscissa. Based on the fitted curve and formula Ka = [Ag/Ab]/([Ag][Ab]), the value of Ka was calculated, and the average Ka values of rSPG-RFP were obtained.

The fluorescence activity of the rSPG-RFP expressed in the bacterial cells was observed under a luminescence microscope. Bacterial cells were collected before IPTG induction and 1 h, 2 h, 4 h, 6 h and 8 h after induction by IPTG. The difference in fluorescence in the bacterial cells was observed over time before and after induction of the expression of rSPG-RFP.

A steady-state transient fluorescence spectrometer (FLS1000, Edinburgh Instruments, UK) was used to analyze the fluorescence characteristics of the recombinant protein rSPG-RFP. The excitation spectrum was scanned to confirm the excitation wavelength with the highest intensity. Subsequently, the emission spectrum was obtained with the confirmed excitation wavelength.

### Establishment of the FICA strip

**Fig. 1:**
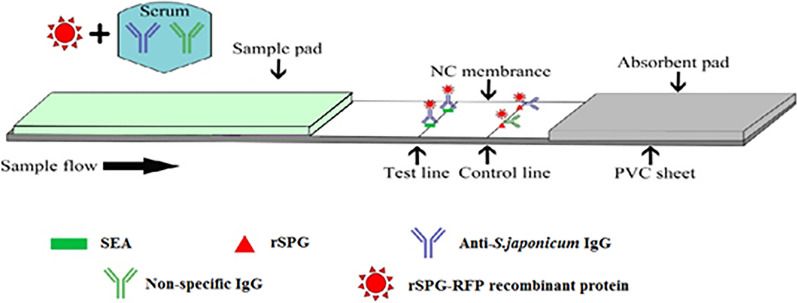
Schematic illustration of the FICA strip

The FICA strip was assembled by using the method presented by RuiXu. Acting as coating antigens in the testing line and control line, SEA and rSPG were expressed according to RuiXu’s steps [12]. The CN95 nitrocellulose membrane worked as a chromatography substrate, and the sample pad was built with a glass cellulose membrane. The sample pad, PVC plate and water-absorbing paper were assembled on the FICA strip. The principle and a schematic illustration of the FICA strip are shown in Fig. 1.

The mixed serum and rSPG-RFP recombinant protein were loaded onto the sample pad. The schistosome SEA was immobilized as the test line in the NC membrane. rSPG was used as the control line. Following the application of a serum sample containing the specific anti-*S. japonicum* IgG and a nonspecific IgG onto the NC membrane, the conjugated anti-*S. japonicum* IgG complex was captured by the SEA on the test line (T), resulting in a fluorescent band. The conjugated anti-*S. japonicum* IgG and nonspecific IgG were captured by the rSPG on the control line (C), resulting in another fluorescent band.

### Comparison of ELISA and FICA strip detection in buffalo serum

The sensitivity of the FICA strips was verified using standard negative serum samples from buffaloes with negative IHA results from *S. japonicum* non-endemic areas. Buffalo serum samples were considered standard positive when *S. japonicum* miracidia were present in the host stool 60 days post-infection.

Standard buffalo *S. japonicum*-positive and *S. japonicum*-negative sera were diluted in a gradient from 1:100 to 1:20,000, and the recombinant protein rSPG-RFP was added. After mixing the samples, they were transferred to the sample pad and exposed under UV light to confirm fluorescence after 5 min. The dilution with detectable fluorescence was considered as the sensitivity of the strip.

The samples were also examined using ELISA to compare the sensitivity and coincidence rate. The wells of the microtiter plates were coated with SEA diluted with carbonate-bicarbonate buffer (pH 9.6) and incubated overnight at 4 °C. Then, the wells were blocked with 5% nonfat milk/PBST for 2 h at 37 °C. Subsequently, the serum samples diluted 1:100 with PBST were added to the wells and incubated for 2 h at 37 °C. After washing with PBST, HRP-conjugated IgG diluted 1:2000 with PBST was added to the wells, and the plates were incubated at 37 °C for 1 h. Then, the plates were washed with PBST, 3,3′,5,5′-tetramethylbenzidine dihydrochloride was added to each well, and the reaction was stopped with the addition of 2 M sulfuric acid. The OD at 450 nm was measured using a microplate reader (Tecan, Mannedorf, Switzerland). A *P*/*N* value ≥ 2.1 was considered positive. We hypothesized that there was a significant difference between indirect ELISA and the FICA strip. This hypothesis will be validated by data analysis.

### Cross-reactivity of FICA and ELISA

Forty-eight goat serum samples positive for *S. turkestanicum* and 63 goat serum samples positive for *H. contortus* were used to evaluate the cross-reactivity of the FICA strips.

### Data analysis

Sensitivity and specificity were assessed by using the following formulas: sensitivity = number of true positives/(no. of true positives + false negatives) and specificity = no. of true negatives/(no. of false positives + true negatives). SPSS 16.0 statistical software was used, and *P* < 0.05 was considered statistically significant. Chi-square tests were used to determine the statistical significance of the differences between ELISA and FICA strips.

## Results

### Cloning and expression of the recombinant plasmid

**Fig. 2:**
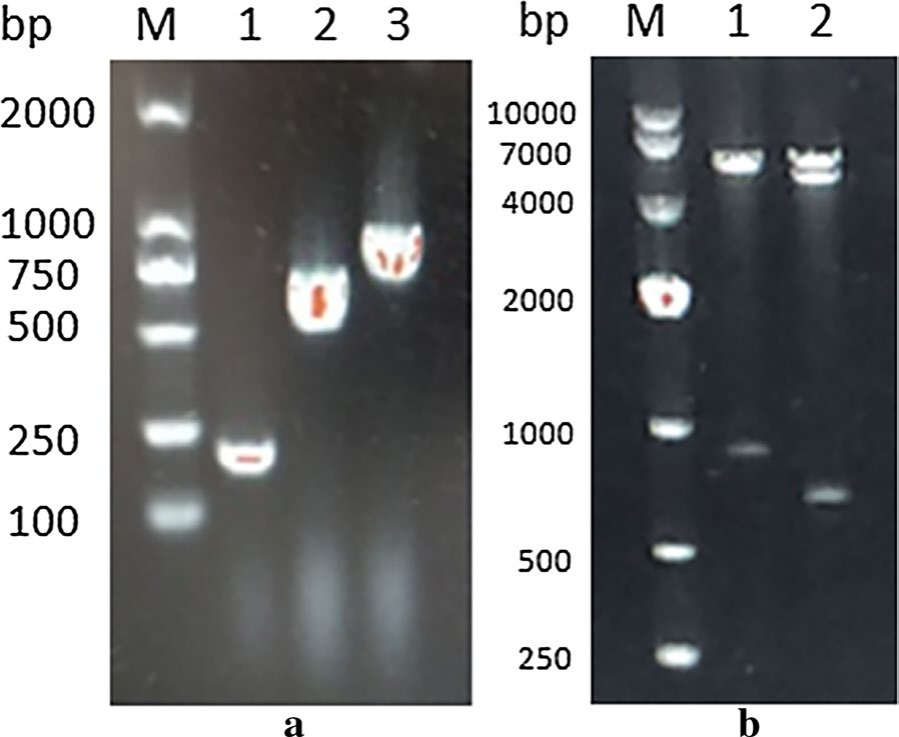
PCR- and enzyme digestion-based validation of the recombinant plasmid. **a** PCR-based validation of the recombinant plasmid. M: DNA marker; lane 1: C3 domain (208 bp); lane 2: RFP fragment (688 bp); lane 3: C3-RFP fragment (896 bp). **b** Double-enzyme digestion-based validation of the recombinant plasmid. Lane 1: C3-RFP sequences obtained by double-enzyme digestion with BamH1 and Xhol1, and the fragment size was 896 bp; lane 2: RFP sequences were obtained by double-enzyme digestion with EcoR1 and Xhol1, and the fragment size was 688 bp

The prokaryotic expression plasmid PET-28a(+)-C3-RFP was constructed by PCR and double-enzyme digestion (Fig. 2). After analyzing the sequence of the correct recombinant plasmid, the result showed that the constructed prokaryotic expression plasmid contained the target genes, among which the C3 domain size was approximately 208 bp, the RFP fragment size was approximately 688 bp, and the full-length fragment size was approximately 896 bp. The sequencing results were consistent with the designed sequence.

**Fig. 3:**
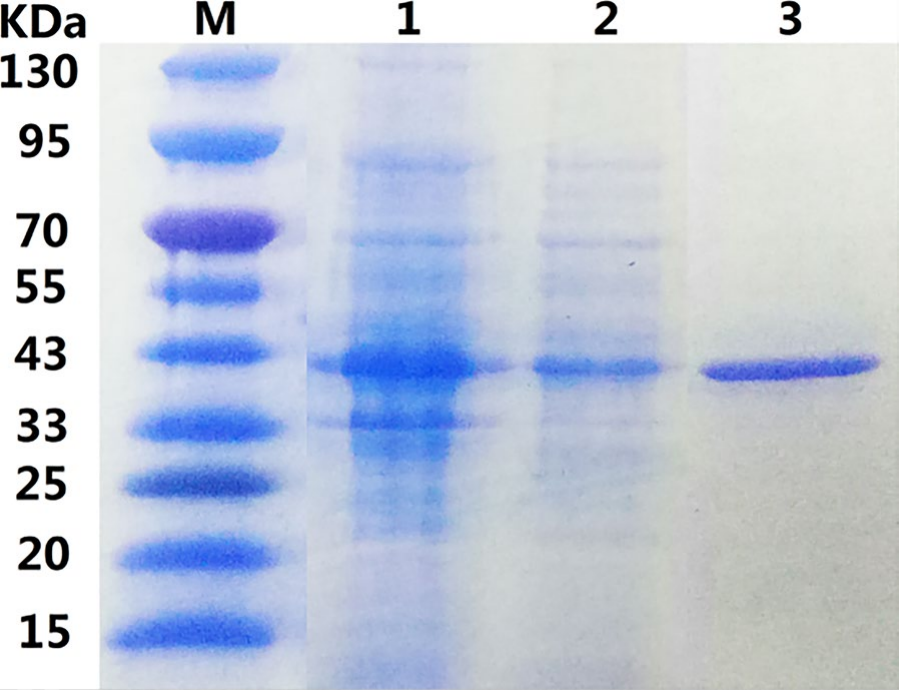
Soluble protein identification and purification analysis of rSPG-RFP. M: marker; lane 1: precipitate; lane 2: supernatant; lane 3: purified protein

Soluble identification and purification analysis of the expressed recombinant proteins was performed. As the SDS-PAGE result shows, the recombinant protein was present in both the soluble and insoluble fractions of the bacterial extract. The soluble fraction was purified with Ni-NTA His Bind Resin (Merck-Millipore, USA), and a specific band appeared at 39.72 kDa, which was consistent with the theoretical molecular weight of the recombinant protein rSPG-RFP (Fig. 3).

### Analysis of the binding activity of the recombinant protein rSPG-RFP with IgG

**Fig. 4:**
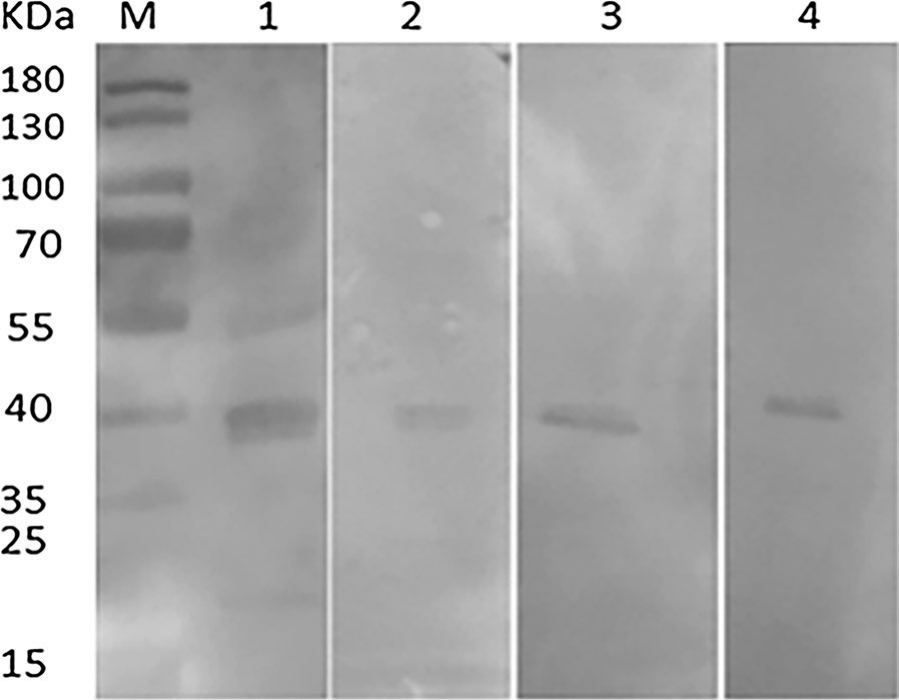
Western blot analysis of rSPG-RFP; M: protein marker; lane 1: purified rSPG-RFP recognized with HRP-conjugated goat IgG; lane 2: purified rSPG-RFP recognized with HRP-conjugated donkey IgG; lane 3: purified rSPG-RFP recognized with HRP-conjugated mouse IgG; lane 4: purified rSPG-RFP recognized with HRP-conjugated rabbit IgG

**Fig. 5:**
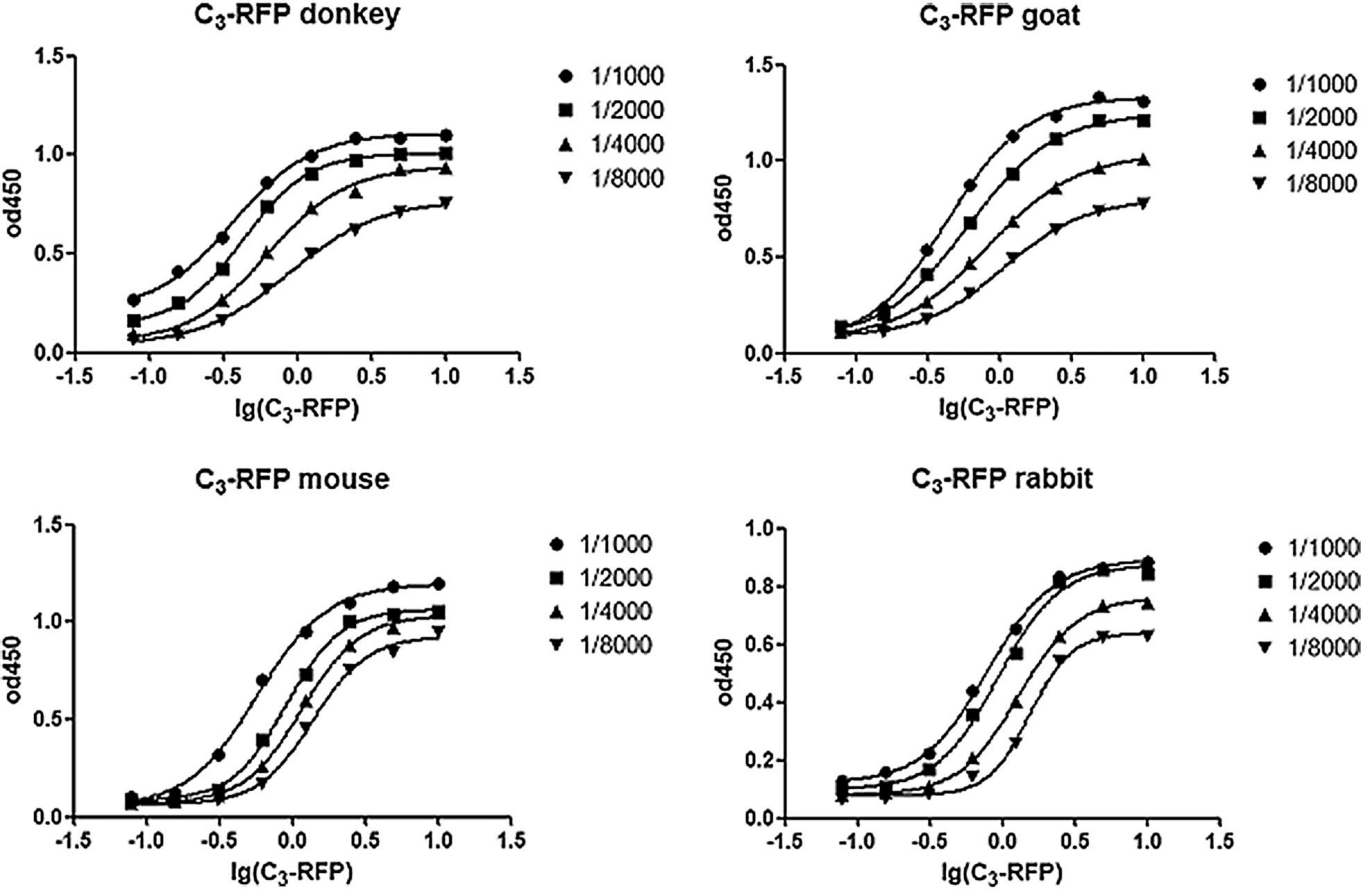
Determination of the Ka of rSPG-RFP with IgG from different animals

The binding activity of the recombinant protein rSPG-RFP with HRP-conjugated multispecies IgG was determined qualitatively and quantitatively by western blot analysis and ELISA. The recombinant protein had the ability to bind with rabbit, donkey, mouse and goat IgGs (Fig 4), and the affinity constant (Ka) values were $1.9 \times 10^{5} ,\; 4.1 \times 10^{5} , \;1.7 \times 10^{5} ,\;{\text{and}}\; 5.4 \times 10^{5}$, respectively (Fig. 5, Table 2).

**Table 2:** Ka of rSPG-RFP with IgG from different animals

Host	Affinity constant
Rabbit	1.9 × 10^5^
Donkey	4.1 × 10^5^
Mouse	1.7 × 10^5^
Goat	5.4 × 10^5^

### Fluorescence activity and characteristics of rSPG-RFP

**Fig. 6:**
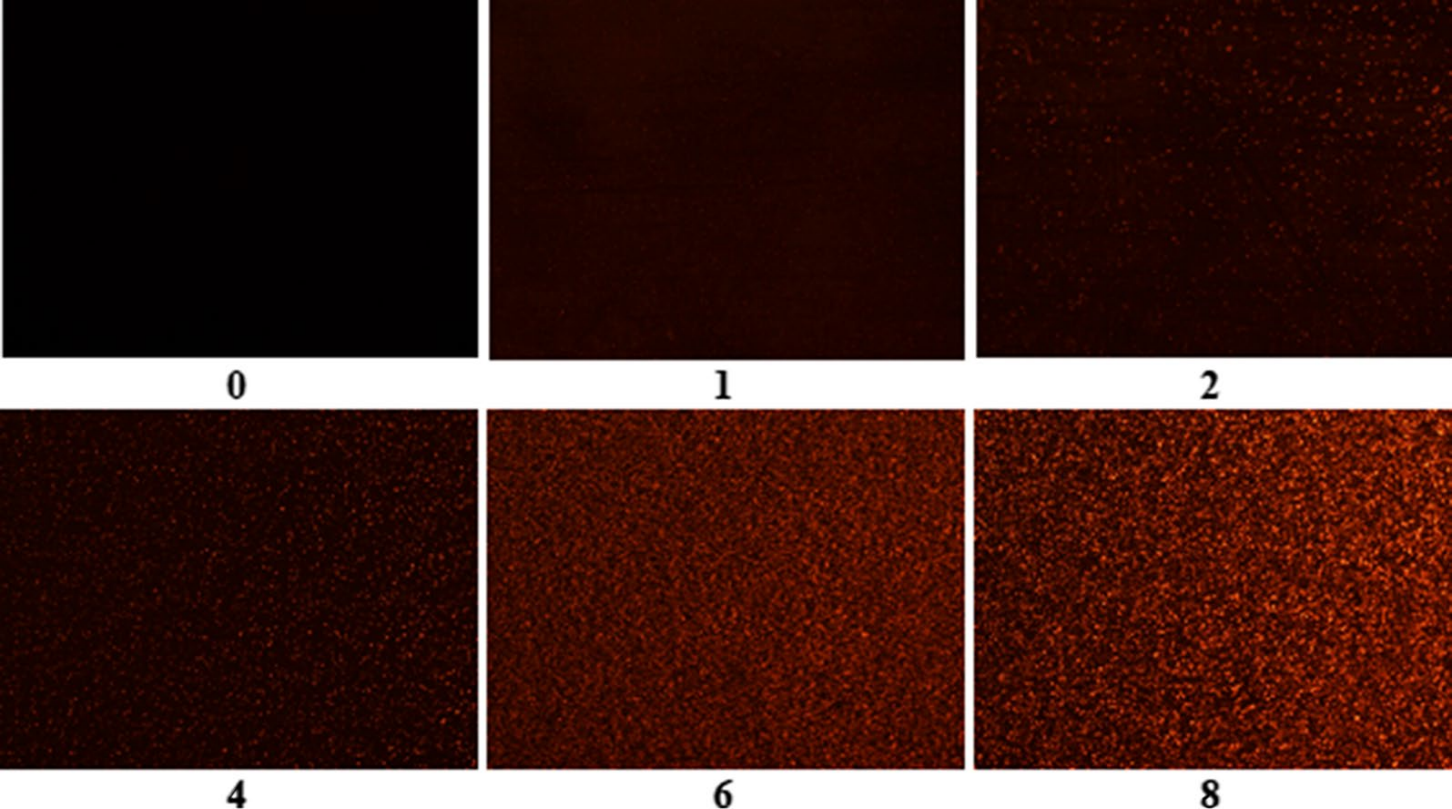
Fluorescence detection of rSPG-RFP expressed in bacteria. 0: without IPTG induction; 1–8: 1 h, 2 h, 4 h, 6 h and 8 h after induction by IPTG

The fluorescence activity of the recombinant protein rSPG-RFP was studied. The fluorescence intensity inside the bacterial cells varied and was monitored by fluorescence microscopy (Fig. 6). After induction by IPTG, red fluorescence gradually appeared inside the bacterial cells, and the fluorescence intensity was proportional to the expression time.

**Fig. 7:**
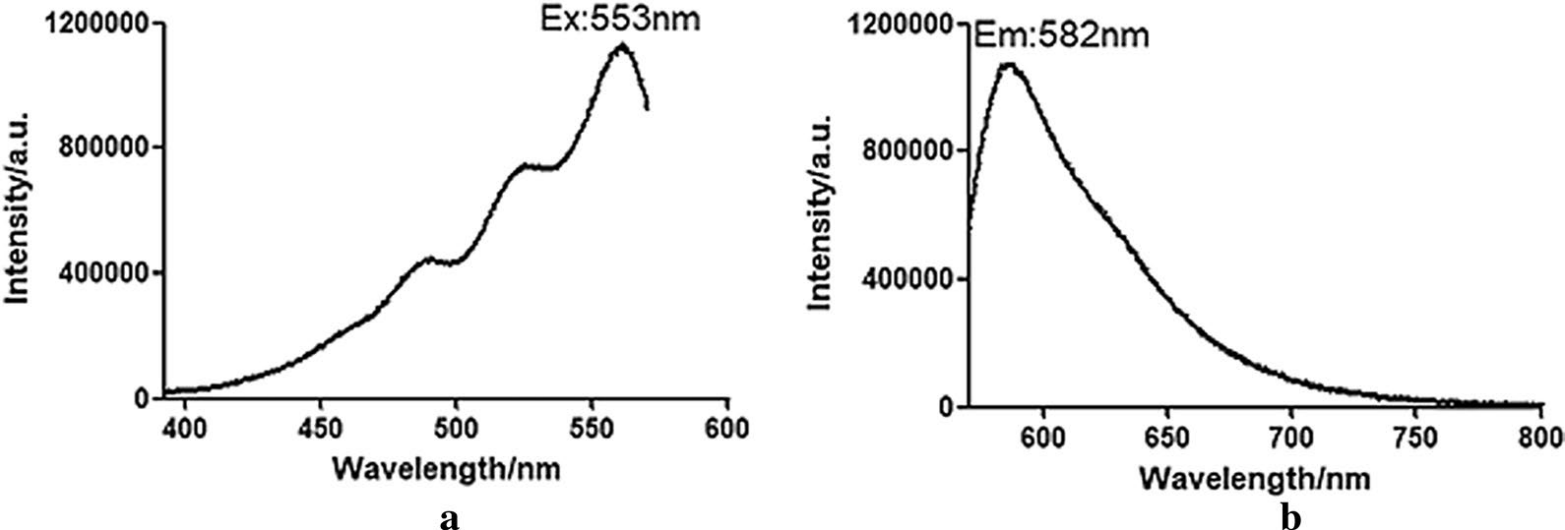
Fluorescence spectrum of the recombinant protein rSPG-RFP at 553 nm. **a** Excitation spectrum; **b** emission spectrum

The fluorescence properties of the recombinant protein rSPG-RFP were analyzed. A fluorescence spectrophotometer was employed to detect the excitation and emission spectra of the recombinant protein. The maximum fluorescence excitation wavelength (*E*_x_) was 553 nm, and the maximum fluorescence emission wavelength (*E*_m_) was 582 nm, which is consistent with literature reports (*E*_x_ = 555 nm/*E*_m_ = 583 nm). The fluorescence intensity of the recombinant protein changed under the optimal excitation light, as shown in Fig. 7.

### Establishment of the FICA strip

**Fig. 8:**
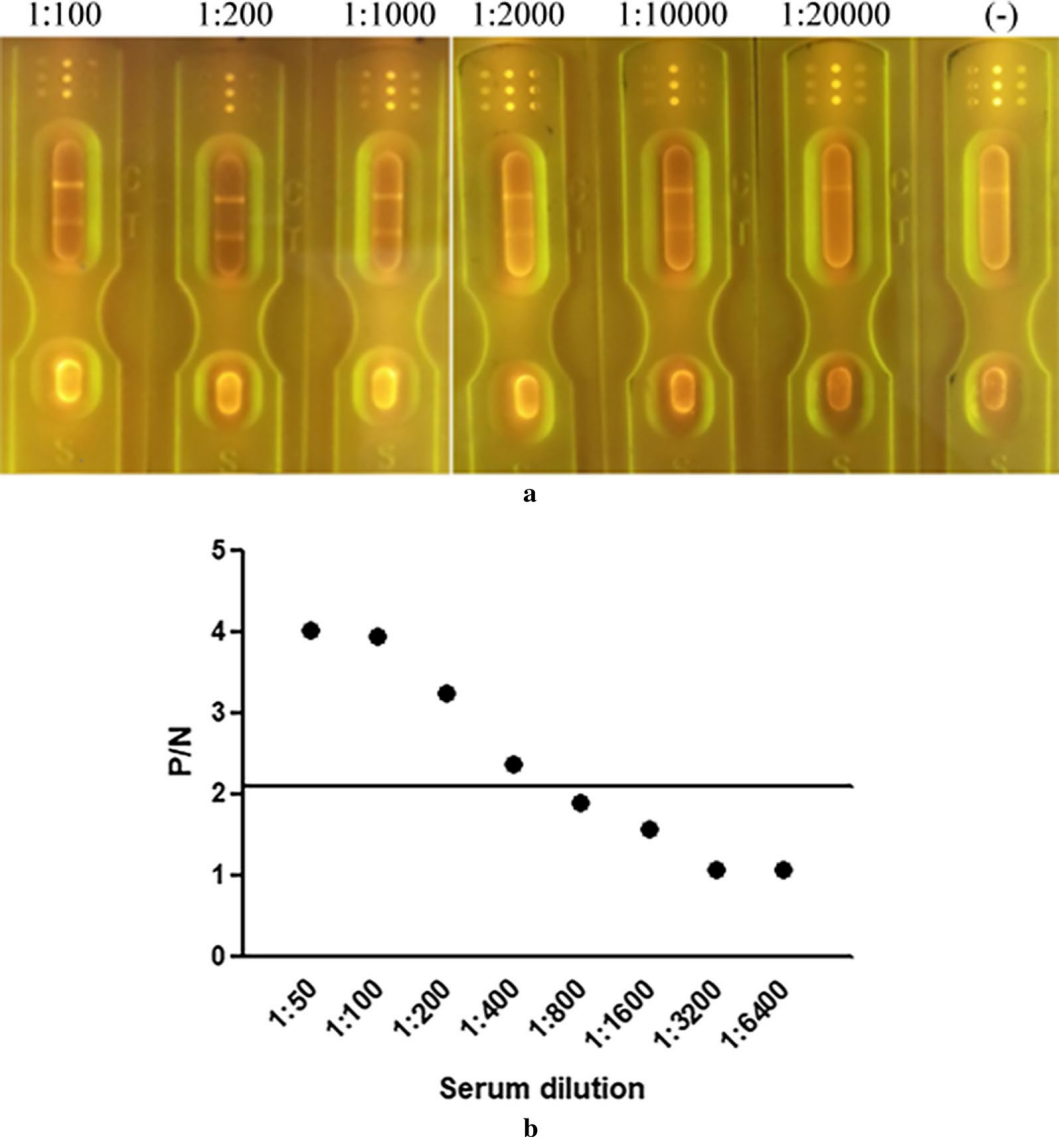
Determination of the sensitivity. **a** Sensitivity of detection by the FICA strips. The *S. japonicum*-positive serum was diluted at 1:10, 1:200, 1:1000, 1:10,000 and 1:20,000; (−) indicates the negative serum that was diluted at 1:100. **b** Sensitivity of detection by ELISA. The *S. japonicum*-positive serum was diluted at 1:50, 1:100, 1:200, 1:400, 1:800, 1:1600, 1:3200 and 1:6400

The SEA of *S. japonicum* and SPG were transferred at a speed of 4 µl/cm at a concentration of 0.5 mg/ml and fixed on a cellulose nitrate film (CN95) as the detection line (T) and quality control line (C), respectively. The FICA strips were assembled with a glass cellulose membrane, PVC bottom plate and absorbent paper. The paper strips were used for the detection of schistosomiasis antibodies (Fig. 8).

All buffalo serum samples were diluted in the range of 1:100 to 1:20,000. The recombinant protein was subsequently added, followed by thorough mixing. The fluorescence intensity was observed under UV light. For ELISA-based detection, *S. japonicum*-positive bovine serum can be diluted to a maximum dilution of 1:400. The results will be negative (*P*/*N* < 2.1) if the serum dilution exceeds 1:400.

### Comparison of ELISA- and FICA strip-based detection in bovine serum

Indirect ELISA and FICA strips were used to detect clinical bovine serum samples. The coincidence rate between FICA strips and indirect ELISA was compared. As shown in Table 3, all 127 positive serum samples tested positive with the FITC strip, whereas 126 of these samples tested positive with ELISA. Among the 82 negative serum samples, 79 serum samples tested negative with FICA strips, and 74 serum samples tested negative with ELISA. Table 4 shows the results of the detection of bovine serum with different infection intensities. The infection intensities were divided into three grades: > 1000, from 100 to 1000 and < 100. When the *S. japonicum* infection intensity was > 1000, all the positive serum samples were judged correctly with both methods. When the *S. japonicum* infection intensity was 100 to 1000, all the positive bovine serum samples were judged correctly as well. When the *S. japonicum* infection intensity was < 100, one case was missed by ELISA detection, and the sensitivity was 96.15%. According to the chi-square test, it was hypothesized that there was a significant difference between indirect ELISA and the FICA strip (*χ*^2^ = 30.29, df = 1, *P* < 0.01) indicating that the null hypothesis was not true and that the difference between the two methods was not significant. Moreover, kappa = 0.9660 indicated good consistency between the two methods. Fecal examination results were used as the gold standard. The sensitivity of the FICA strip was 100%, and the specificity was 96.34%.

**Table 3:** Comparison of ELISA and FICA strips

	No. of positive	Positive rate (%)	No. of negative	Negative rate (%)
FICA strip	127	100.00	79	96.34
ELISA	126	99.21	74	90.24

**Table 4:** Detection of sera with different infection intensities

Infection degree	FICA strips		ELISA	
	No. of positive	Positive rate (%)	No. of positive	Positive rate (%)
≥ 1000	72/72	100.00	72/72	100.00
100–1000	29/29	100.00	29/29	100.00
< 100	26/26	100.00	25/26	96.15

### Cross-reactivity of the FICA strips

The cross-reactivity of the FICA strips is shown in Table 5. The cross-reaction of the FICA strips with *S. turkestanicum* in goats was 91.66% (44/48), which was significantly higher than that of ELISA (87.50%, 42/48). Moreover, the cross-reactivity of the FICA strips with *H. contortus* in goats was 30.15% (19/63), which was higher than that of ELISA (26.98%, 17/63).

**Table 5:** Cross-reactivity of FICA strips and ELISA

Serum	No. of cases	FICA		ELISA	
		No. of positive	Positive rate (%)	No. of positive	Positive rate (%)
Serum from *Schistosoma turkestanicum*-positive goat	48	44	91.66	42	87.5
Serum from *Haemonchus contortus*-positive goat	63	19	30.15	17	26.98

## Discussion

RFP is a bioluminescent protein isolated from mashroom coral. It has a relatively high emission wavelength and can avoid interference from spontaneous green fluorescence in living creatures [13, 14]. The protein contains 248 amino acids, and it emits more intense fluorescence than green fluorescent protein (GFP) mutants. Moreover, RFP exhibits strong penetration ability, and the signal lasts for a long duration. In the infrared spectrum, RFPs show diversity among fluorescent proteins [15]; however, most RFPs require special excitation light sources such as ultraviolet light [16–24]. DsRed2 is an artificial mutant gene derived from wild-type DsRed that expresses RFP2. Its protein structure is similar to that of GFP. In addition, it emits red fluorescence under natural light, which gives it the potential to become a new reporter gene.

**Fig. 9:**
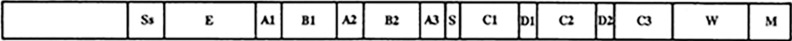
Structure of SPG

SPG is a streptococcal cell wall protein with the ability to bind to a variety of human and animal IgG antibodies. This protein was first reported in 1973 by Kronvall [25]. Later, in 1984, Bjorck named, separated and purified SPG [26]. Compared with staphylococcal protein A (SPA), which is more commonly used in colloidal GICA strips, SPG exhibits higher affinity for IgG binding and has a wider range of applications [27], and it is of great importance for the detection and purification of antibodies as an alternative to SPA [28–32]. Some regions of homology have been reported in the structure of SPG (Fig. 9). The C domain (containing domains C1, C2 and C3) of SPG at the C-terminus has been noted to affect the binding of SPG to the IgG Fc region. While the C1 and C2 domains differ in only two amino acids, the C1 and C3 domains differ in six amino acids. The IgG-binding capacity of the C3 domain has been found to be seven times higher than that of the C1 domain [33].

In this study, a fusion protein consisting of IgG-binding domains of SPG (C3 domain) and RFP was constructed. The protein rSPG-RFP retained both activities: the IgG-binding capability of SPG and the enzymatic activity of RFP. The affinity constant of rSPG-RFP with IgG from different animals was quite different from that of the reported SPGs. The reason for this result was that the protein that we expressed possesses only one C3 domain that binds with multispecies IgG. Xu et al. [12] constructed recombinant proteins with different structures (C3DC3 and C3DC3DC3) and found that the binding ability of the protein with multispecies IgG relates to the number of C3 domains. The rSPG-RFP that we developed contained only one C3 domain, which resulted in a low affinity constant, which is consistent with the conclusions of the previous studies.

We also found that the fluorescence intensity of rSPG-RFP began to increase significantly 4 h after expression and had increased several fold by 8 h. The fluorescence intensity of rSPG-RFP under 532 nm excitation light and 582 nm reflected light showed no significant difference from that of RFP, which suggested that the recombinant protein rSPG-RFP folded correctly and that the recombinant expression of the two genes did not affect the function of rSPG-RFP.

When the recombinant protein was applied for FICA strip detection, the IgG in the serum to be measured was combined with rSPG-RFP to form labeled IgG. IgG that specifically bound to SEA did so on the test line, while nonspecific IgG bound to SPG on the control line. Under ultraviolet light, red fluorescence signals can be distinguished by the naked eye. This method has higher sensitivity than ELISA, which is reflected in the serum dilution rate. The highest dilution rate of bovine *S. japonicum*-positive serum detected by ELISA was 1:400. In contrast, the FICA strips established in this study gave positive results at a 1:10,000 serum dilution, showing a higher sensitivity.

When the FICA strips and indirect ELISA were simultaneously used to detect the *S. japonicum*-positive or *S. japonicum*-negative sera, it was found that the above two methods showed basically the same test results when detecting *S. japonicum*-positive sera of buffaloes infected with more than 100 schistosomes. The positivity rate for both methods was 100%.

When the number of *S. japonicum* in buffaloes was < 100, the ELISA result was negative in one case, and the detection rate was 96.15%. In contrast, the detection rate of the FICA strip remained 100%. Although the difference in sensitivity of the two methods is not obvious, this result implies that FICA strips, compared with ELISA, are more suitable for detecting samples with low infection intensities. Based on the detection rates for all the above-mentioned positive sera with different intensities of *S. japonicum* infection, the positivity rate of the ELISA method was 99.21%, and the positivity rate of the fluorescent strip method was 100%. Therefore, the detection results of the two methods were basically consistent (kappa = 0.9660). Among the 82 *S. japonicum*-negative buffalo serum samples, 74 tested negative by ELISA, showing a specificity rate of 90.24%. The FICA strip showed 79 negative cases, and the specificity rate was 95.12%. Therefore, compared to the ELISA method, the sensitivity and specificity of the fluorescent immunoassay strips were 100% and 96.34%, respectively, showing the high practical value of this technique.

However, the cross-reactivity of FICA strips with *H. contortus* and *S. turkestanicum* (30.15% and 91.66%, respectively) was higher than that of ELISA (26.98% and 87.5%, respectively). There was some cross-reactivity with other parasitic antigens, especially for FICA, which exhibited high cross-reactivity with *S. turkestanicum* in goats. Whether a more specific diagnostic antigen is needed was considered from two aspects. First, the epidemic areas are different between *S. japonicum* and *S. turkestanicum*. Second, SEA was the antigen for which the sensitivity was the highest when diagnosing schistosomiasis japonica, and other available diagnostic antigens were also used. The greatest utility of this strip was in early screening, and it can achieve its purpose in combination with other diagnostic methods. For various policy restrictions, we have no access to obtaining serum samples with *S. mansoni*, *S. haematobium*, *S. mekongi*, *Paragonimus* species, *Clonorchis* species or hepatitis B virus antibodies. However, we will carry out the cross-reaction test of FICA strips with these species as long as we obtain the serum.

With the binding characteristics of IgG and the fluorescence characteristics of RFP, the recombinant protein rSPG-RFP was used to verify the application of the FICA strips based on this protein. rSPG-RFP has promising potential applications in the establishment of test strips for detection.

## Conclusions

The recombinant protein rSPG-RFP was successfully constructed and expressed, and the FICA strip for schistosomiasis japonica diagnosis based on rSPG-RFP was established. The sensitivity was higher than that of indirect ELISA, and the detection result was basically consistent with that of the indirect ELISA method (kappa = 0.9660, *P* < 0.01). The sensitivity and simplicity of its application make this method suitable for large-scale screening.

## Data Availability

The datasets used or analyzed during the current study are available from the corresponding author on reasonable request.

## Abbreviations

FICA strip: Fluorescence immunochromatographic assay strip
SPG: Streptococcal protein G
RFP: Red fluorescent protein
ELISA: Enzyme-linked immunosorbent assay
*S. japonicum*: *Schistosoma japonicum*
IHA: Indirect haemagglutination assay
SEA: Soluble egg antigen
